# Associations of wearable photobiomodulation device use with mitochondrial bioenergetics and fatigue in athletes and individuals with chronic fatigue

**DOI:** 10.3389/fspor.2026.1823212

**Published:** 2026-07-02

**Authors:** Zulia Valeyeva-Frost, Greg Eckel

**Affiliations:** 1 Clinical Department, Recharge Health, Oslo, Norway; 2 Clinical Research Site, Park City, UT, United States

**Keywords:** athletes, ATP production, cellular bioenergetics, chronic fatigue, glycolysis, mitochondrial function, near-infrared light, photobiomodulation therapy (PBMT)

## Abstract

This 30-day observational pilot study evaluated associations between wearable photobiomodulation use and changes in mitochondrial bioenergetics and perceived fatigue in two physiologically distinct cohorts: athletes (*n* = 10) and individuals with chronic fatigue (*n* = 10). Participants used a dual-wavelength LED photobiomodulation device six days per week, performing four sequential 10-minute placements per session. Mitochondrial function was assessed at baseline (Day 0), Day 15, and Day 30 using the validated meScreen™ bioenergetic assay from finger-prick blood samples. Subjective fatigue was measured using the FACIT-10a in the chronic fatigue cohort and the MFI-20 in athletes. Within-group changes were analyzed using non-parametric Wilcoxon signed-rank tests. By Day 15, early bioenergetic shifts were observed. The chronic fatigue cohort demonstrated significant increases in baseline respiration (*p* = 0.0195) and mitochondrial potential (*p* = 0.0352), consistent with changes in mitochondrial reserve capacity. Athletes demonstrated significant increases in mitochondrial efficiency (*p* = 0.0195) and reduced glycolytic reliance (*p* = 0.0488), consistent with shifts in metabolic efficiency rather than increased total energy output. By Day 30, these directional changes were largely maintained. Mitochondrial potential showed a non-significant trend toward increase across cohorts (*p* = 0.0774). No increases in reactive oxygen species or mitochondrial network instability were observed at any timepoint. Subjective fatigue scores decreased by 37% in the chronic fatigue cohort and 12% in athletes, with approximately 63% of total reduction occurring within the first 15 days. Thirty days of wearable photobiomodulation use was associated with directional changes in selected bioenergetic parameters alongside reductions in perceived fatigue. These findings support further controlled studies to evaluate durability, dose–response relationships, and clinical relevance.

## 1 Introduction

Fatigue is a highly prevalent and clinically significant symptom observed across medical, occupational, and athletic populations. Population-based studies estimate that approximately 20%–30% of adults report persistent fatigue, with chronic fatigue defined as fatigue lasting longer than six months, affecting approximately 10% of the adult population (1). Epidemiological analyses further demonstrate a female-predominant distribution and distinguish between explained and unexplained fatigue presentations.

Importantly, chronic fatigue as a symptom descriptor should not be conflated with myalgic encephalomyelitis/chronic fatigue syndrome (ME/CFS), which represents a distinct clinical diagnosis defined by specific criteria and substantially lower prevalence estimates. The present study focuses on fatigue as a functional and physiological construct rather than on diagnostic classification.

In athletic populations, fatigue is a key feature of overtraining syndrome (OTS), and it is estimated that between 20% and 60% of athletes experience it at least once during their careers (2). Despite its prevalence and impact, fatigue remains one of the most challenging physiological constructs to quantify objectively due to its inherently subjective and multidimensional nature.

One of the primary challenges in fatigue impact is the absence of a single, definitive biomarker. Fatigue is a multidimensional concept encompassing physical, cognitive, and motivational components, influenced by metabolic, neurological, endocrine, and psychosocial factors (3). As a result, fatigue assessment has traditionally relied on subjective patient-reported outcome measures. There are various tools to measure a broad range of fatigue dimensions: physical, cognitive, mental, central, peripheral, emotional, motivational, and psychosocial. The three most widely used questionnaires to measure physical fatigue were the Multidimensional Fatigue Inventory (MFI), the Modified Fatigue Impact Scale (MFIS), and the Multidimensional Assessment of Fatigue (MAF). Motivational fatigue specifically used with fibromyalgia patients: the Multidimensional Daily Diary of Fatigue-Fibromyalgia-17 (4) and the Patient-Reported Outcomes Measurement Information System (PROMIS) Fatigue Fibromyalgia Profile (5). For assessment of psychological impact other questionnaires are used, such as Borg scale and the Functional Assessment of Chronic Illness Therapy-Fatigue (FACIT-F) questionnaire (6). FACIT-10a is a validated fatigue short form derived from the FACIT fatigue item bank and psychometrically harmonized with the PROMIS Fatigue framework. While these instruments are validated and clinically meaningful, they capture perception rather than underlying biological mechanisms.

In this study, two validated fatigue assessment tools were selected: the Functional Assessment of Chronic Illness Therapy–Fatigue short form (FACIT-10a) and the Multidimensional Fatigue Inventory (MFI-20), based on their brevity and acceptability to participants.

In a search for a biological indicator to evaluate fatigue, we investigated a primary mechanism, cellular mitochondria functioning. At a cellular level, fatigue is increasingly understood as a manifestation of impaired energy production and utilization, with mitochondria playing a central role. Mitochondria are responsible for aerobic ATP production, regulation of redox balance, calcium handling, and metabolic signaling. Mitochondrial dysfunction has been implicated in chronic fatigue syndromes, neuroinflammatory conditions, metabolic disease, aging, and impaired exercise recovery (7). Importantly, mitochondrial dysfunction does not imply reduced mitochondrial number; rather, deficits often arise from reduced efficiency, diminished reserve capacity, impaired stress responsiveness, or maladaptive metabolic shifts (8).

Despite this recognition, measuring mitochondrial function in humans presents substantial methodological challenges. The historical gold standard for mitochondrial assessment such as muscle biopsy (9) followed by high-resolution respirometry or electron microscopy is invasive, painful, and impractical for repeated or large-scale studies, these tests are primarily focused on detection of rather rare incidences of mitochondrial disorders. Moreover, biopsy-based approaches are tissue-specific, limited in ecological validity, and primarily suited for mechanistic laboratory investigations rather than longitudinal human studies.

Alternative approaches, such as indirect metabolic and genetic profiling (10), functional assays and even advanced imaging and spectroscopy offer inferential insight into cellular metabolism but do not directly measure mitochondrial performance under stress or dynamic demand. These tests are costly, analytically complex, and have their own limitations. As a result, there remains a critical gap between subjective fatigue assessment and objective, functionally relevant mitochondrial biomarkers.

Recent advances in cellular bioenergetics have enabled the development of non-invasive, repeatable assays that evaluate mitochondrial function in living cells obtained from peripheral blood samples (11, 12). This study utilized a comprehensive analysis that measures mitochondrial efficiency through a 12-matrix panel, accurately quantifying basal reactive oxygen species levels under high stress and analyzing cellular images for mitochondrial structure, integrity, and organization, offering a more physiologically meaningful representation of cellular energy health. Scientifically validated methods provide an insight into mitochondrial parameters such as baseline respiration, mitochondrial efficiency, reserve capacity, glycolytic reliance, oxidative stress, and network stability under controlled laboratory conditions.

Photobiomodulation (PBM), utilizing red and near-infrared light, has emerged as a promising intervention capable of modulating mitochondrial function (13, 14). Preclinical studies have demonstrated that PBM can influence cytochrome c oxidase activity, enhance mitochondrial membrane potential, increase ATP synthesis, and regulate reactive oxygen species signaling (15). Early human studies suggest beneficial effects on muscle recovery, pain modulation, inflammation, and fatigue-related outcomes; however, most investigations have relied predominantly on subjective measures or performance endpoints (16–19).

Critically, few human studies have directly linked objective mitochondrial bioenergetic biomarkers with validated fatigue outcomes, particularly across populations with differing baseline physiological states, such as individuals with chronic fatigue vs. trained athletes. This represents a significant gap in the literature, as baseline mitochondrial function is likely to influence both the direction and magnitude of physiological adaptation to PBM interventions (20).

Therefore, the present study aims to evaluate the effects of near-infrared photobiomodulation on mitochondrial function and fatigue outcomes using a validated, non-invasive mitochondrial bioenergetic assay alongside standardized fatigue questionnaires. By integrating objective cellular energy metrics with subjective functional outcomes, this study seeks to clarify whether PBM-associated improvements in fatigue are accompanied by biologically coherent and sustainable mitochondrial adaptations.

## 2 Methods

### 2.1 Study design

This study was a 30-day, single-arm, exploratory pilot investigation conducted using meScreen™ test from Versea Discovery (San Diego, CA). All biospecimen collection and meScreen™ testing were conducted under an IRB-approved protocol (Pro00076660). The study was designed to evaluate changes in mitochondrial bioenergetics and subjective fatigue following daily photobiomodulation exposure.

Two cohorts were enrolled to represent distinct baseline physiological states:

- individuals reporting chronic fatigue, representing a recovery-oriented profile, and
- healthy athletes undergoing regular training load, representing an optimization-oriented profile.

The exploratory design was selected to assess feasibility, signal directionality, and biological coherence across heterogeneous baseline conditions.

### 2.2 Participants

A total of 20 adult participants were enrolled and completed the study:

- Chronic fatigue cohort: *n* = 10, female, average age of 44.9 ± years old
- Athletic cohort: *n* = 10, 3/7 male/female ratio, average age 46.6 ± years old

Participants in the chronic fatigue cohort were defined by persistent self-reported fatigue lasting longer than three months and impacting daily functioning, rather than by formal diagnosis of ME/CFS. Individuals with systemic medical conditions known to cause or confound fatigue, or those undergoing active medical treatment for such conditions, were excluded during screening.

All participants provided written informed consent prior to enrollment. The study procedures did not require venous blood sampling, fasting, or medication washout. Participants were instructed to maintain their usual lifestyle and training routines throughout the study period.

### 2.3 Photobiomodulation exposure protocol

Participants used the FlexBeam wearable LED photobiomodulation device emitting near-infrared light (800–850 nm), a wavelength range within the optical window known for deeper tissue penetration and interaction with mitochondrial chromophores. The device delivered an optical power density of 110 mW/cm^2^, resulting in an energy density of 66 J/cm^2^ over an active treatment area of 81 cm^2^ in direct skin contact. These exposure parameters were selected to ensure delivery within established photobiomodulation dosing ranges, where wavelength, power density, and energy density are known to influence the magnitude and direction of biological response (21).

The FlexBeam protocol was standardized across participants. The device was applied six days per week with one rest day. Each session consisted of four sequential 10-minute applications. During each session, the device was applied sequentially to four anatomical regions, including the posterior spinal region and the anterior chest and abdominal regions, in accordance with manufacturer recommendations.

Participants received standardized written, visual and verbal instructions regarding device use and placement to ensure protocol consistency throughout the study period.

### 2.4 Outcome measures

#### 2.4.1 Mitochondrial bioenergetics

Mitochondrial function was assessed using the meScreen assay, a validated laboratory-based bioassay that evaluates cellular bioenergetics using a proprietary adaptive transfer assay. The assay assesses mitochondrial performance under both basal and stressed conditions, enabling evaluation of dynamic energy metabolism rather than static biomarkers. The meScreen™ platform utilizes peripheral blood samples collected via finger-prick onto a proprietary bilayer card, which enables serum separation and sample stabilization at ambient temperature for up to two months. Samples are reanimated in an optimized buffer and subjected to a 12-matrix bioenergetic panel that integrates functional respirometry with high-content fluorescence imaging and machine-learning-based analysis. The platform has been validated at the University of California, San Diego, against over 60 FDA-approved compounds and probes with known mitochondrial activity, demonstrating sensitivity to detect changes in both mitochondrial function and structural dynamics. Assay reproducibility has been established through repeated measures under standardized laboratory conditions. Reported parameters include baseline respiration, mitochondrial efficiency, reserve capacity, glycolytic reliance, reactive oxygen species production, and mitochondrial network stability.

The following parameters were analyzed:

- Baseline respiration (resting energy production)
- Mitochondrial efficiency (energy leak)
- Mitochondrial potential (reserve capacity)
- Glycolytic score (non-mitochondrial energy reliance)
- Aerobic score (mitochondrial energetic demand)
- Reactive oxygen species (ROS) production
- Mitochondrial network stability

#### 2.4.2 Subjective fatigue

Subjective fatigue was assessed using validated questionnaires appropriate to each cohort. The chronic fatigue cohort completed the Functional Assessment of Chronic Illness Therapy—Fatigue (FACIT-10a), while the athletic cohort completed the Multidimensional Fatigue Inventory (MFI-20), reported as total fatigue score. Assessments were performed at baseline (Day 0), midpoint (Day 15), and endpoint (Day 30). Fatigue was assessed using two different validated instruments in two cohorts (FACIT-10a and MFI-20), which are not intended for direct score comparison.

### 2.5 Statistical analysis

Given the exploratory nature of the study and limited sample size (*n* = 10 per cohort), non-parametric statistical methods were applied. Within-group changes across timepoints were analyzed using Wilcoxon signed-rank tests. No correction for multiple testing was applied due to the exploratory design of this pilot study.

Descriptive statistics are reported as mean  ± SEM. Paired effect sizes were calculated using Cohen's d to characterize the magnitude of within-group changes. Wilcoxon signed-rank tests served as the primary inferential method for all within-group comparisons, given the small sample size and non-normal distribution assumptions. Paired Student's *t*-tests were additionally reported for baseline-to-Day 15 and baseline-to-Day 30 comparisons as supplementary parametric sensitivity analysis; however, all inferential conclusions are based on the non-parametric results.

Accordingly, statistical interpretation emphasized directionality, consistency across related bioenergetic domains, and biological coherence rather than formal hypothesis confirmation or inferential generalization. Effect sizes are reported alongside *p*-values to provide a more complete characterization of observed changes. A comprehensive summary of all statistical tests, *p*-values, effect sizes, and descriptive statistics is provided in Supplementary Table S1. These findings should be considered hypothesis-generating and intended to inform the design of future controlled trials.

## 3 Results

### 3.1 Mitochondrial bioenergetic adaptations

#### 3.1.1 Chronic fatigue cohort

Participants in the chronic fatigue cohort demonstrated measurable improvements in multiple mitochondrial bioenergetic parameters over the 30-day intervention period.

Mitochondrial potential showed a significant increase by Day 15 compared with baseline (*p* = 0.037, *d* = 0.84), with a continued positive trend through Day 30. Directional improvements in mitochondrial efficiency were observed across the study period, stabilizing by the endpoint assessment. In parallel, a directional reduction in glycolytic reliance was noted, indicating a shift away from compensatory, non-mitochondrial energy pathways.

**Figure 1:**
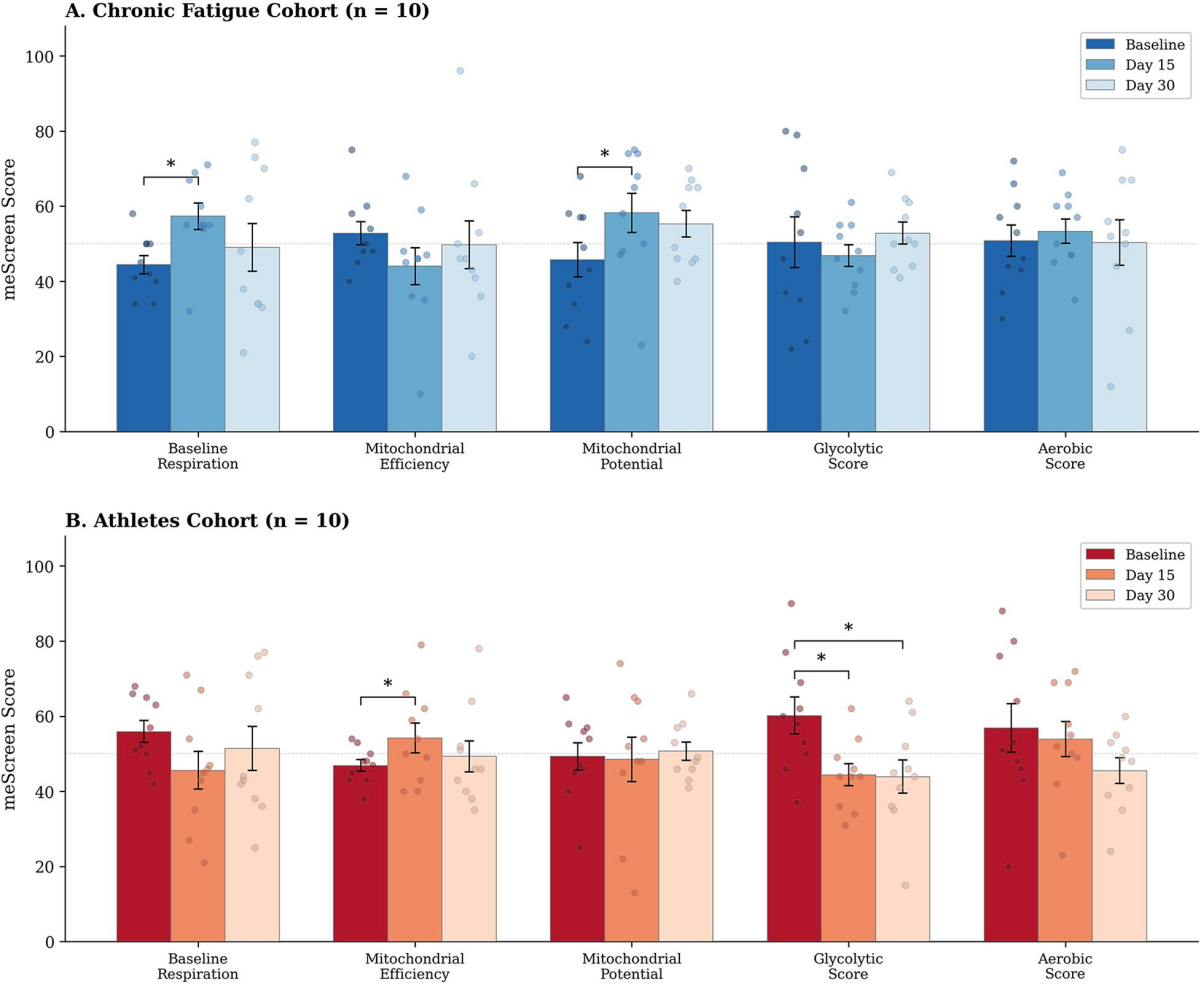
Mitochondrial bioenergetic parameters at Baseline, Day 15, and Day 30 for **(A)** Chronic Fatigue and **(B)** Athletic cohorts. Bars represent group means; error bars indicate ± SEM. Individual participant values are shown as circles. Significance brackets indicate Wilcoxon signed-rank test results: *p < 0.05, **p < 0.01, ***p < 0.001. Dashed line indicates population reference (score = 50).

**Figure 2:**
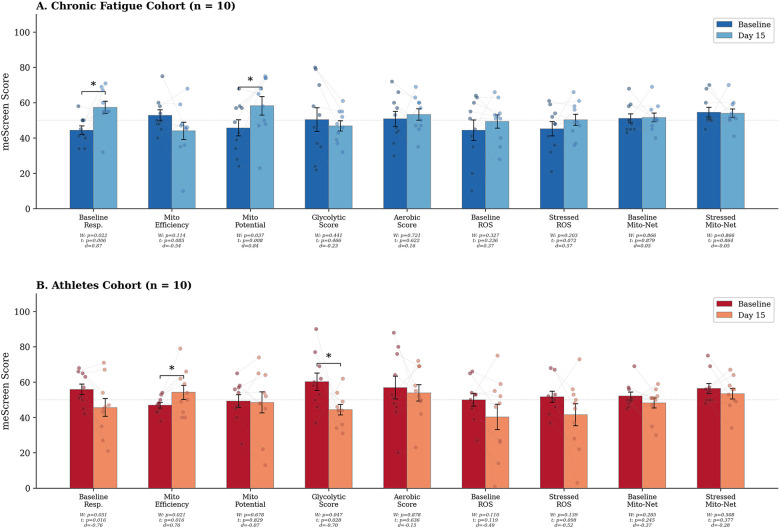
Paired comparison of all bioenergetic parameters: Baseline vs. Day 15. **(A)** Chronic Fatigue cohort; **(B)** Athletic cohort. Bars represent group means ± SEM. Individual participant values shown as circles with grey lines connecting paired observations. Significance brackets: *p < 0.05, **p < 0.01, ***p < 0.001 (Wilcoxon signed-rank test). Both Wilcoxon (W) and paired *t*-test (t) *p*-values shown below each parameter with Cohen's d effect sizes.

**Figure 3:**
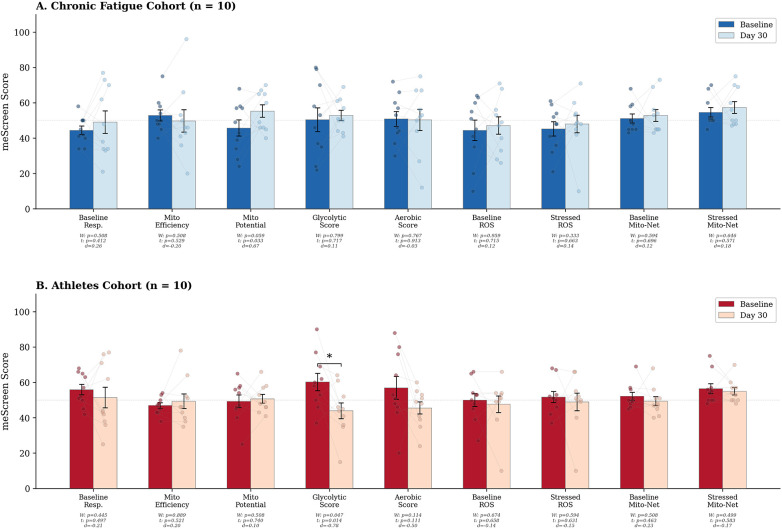
Paired comparison of all bioenergetic parameters: Baseline vs. Day 30. **(A)** Chronic Fatigue cohort; **(B) A**thletic cohort. Bars represent group means ± SEM. Individual participant values shown as circles with grey lines connecting paired observations. Significance brackets: *p < 0.05, **p < 0.01, ***p < 0.001 (Wilcoxon signed-rank test). Both Wilcoxon (W) and paired *t*-test (t) *p*-values shown below each parameter w.

Mean Mitochondrial Potential increased from 45.7 ± 4.6 at baseline to 58.2 ± 5.2 at Day 15 (Cohen's *d* = 0.84, *p* = 0.037). Baseline respiration also increased significantly (44.4 ± 2.4 to 57.3 ± 3.5; *d* = 0.87, *p* = 0.022) (Figure 1A). Pairwise comparisons across all bioenergetic parameters are presented in Figures 2A, 3A; full statistical results are reported in Supplementary Table S1.

**Figure 4:**
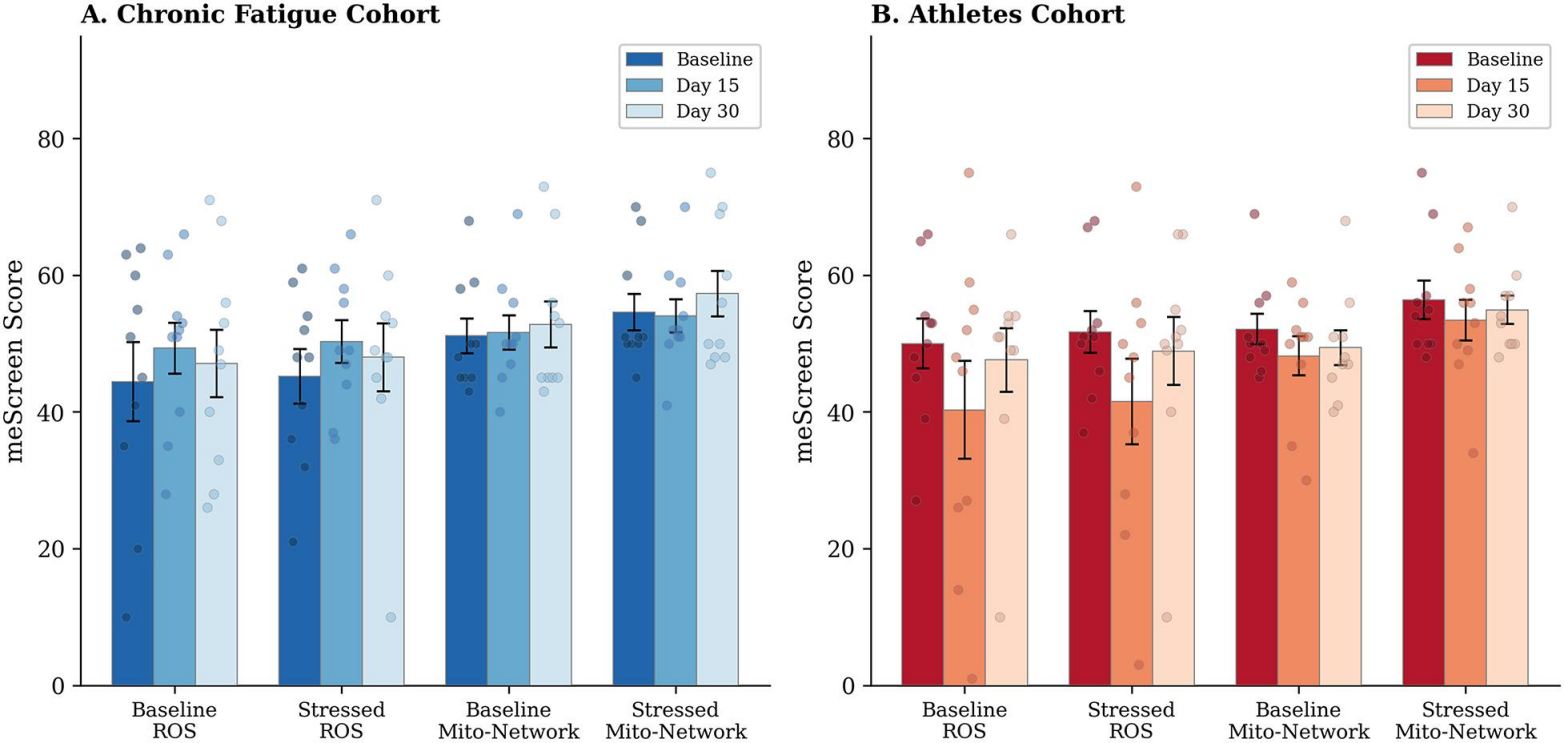
Reactive oxygen generation and Mito-Network Stability at Baseline, Day 15, and Day 30. (**A**) Chronic Fatigue cohort; (**B**) Athletic cohort. Bars represent group means ± SEM with individual participant data shown as circles. No significant changes in ROS or network stability were observed at any timepoint in either cohort (all *p* > 0.05, Wilcoxon signed-rank test).

Importantly, these bioenergetic changes occurred without an increase in reactive oxygen species (ROS) production and without evidence of mitochondrial network instability, suggesting adaptive rather than stress-induced cellular responses (Figure 4A).

**Figure 5:**
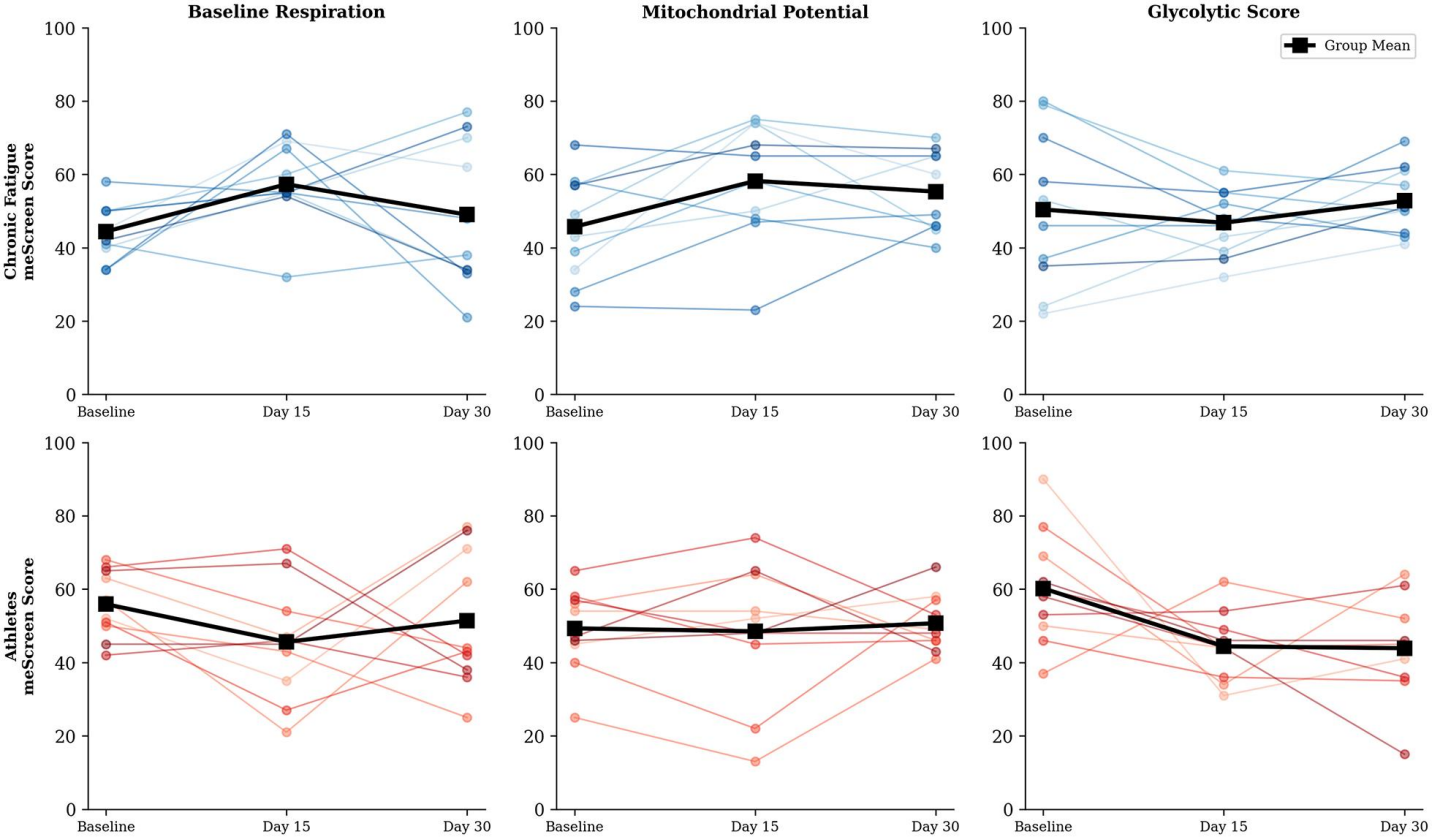
Individual participant trajectories for key bioenergetic parameters across study timepoints. Top row: Chronic Fatigue cohort (*n* = 10); Bottom row: Athletic cohort (*n* = 10). Coloured lines represent individual participants; bold black line with squares indicates the group mean trajectory. Parameters shown: Baseline Respiration, Mitochondrial Potential, and Glycolytic Score.

Collectively, directional changes across mitochondrial reserve capacity, baseline respiration, and glycolytic reliance in the chronic fatigue cohort were associated with large within-group effect sizes (Cohen's *d* ≈ 0.84–0.87 across responsive parameters) (Figure 5). This pattern is consistent with normalization of cellular bioenergetic function from a lower baseline energetic state, reflecting recovery of mitochondrial reserve capacity rather than supraphysiologic upregulation of mitochondrial respiration.

#### 3.1.2 Athletic cohort

Athletes exhibited a distinct pattern of mitochondrial adaptation characterized by efficiency gains rather than increased total energy output.

Mitochondrial efficiency improved significantly by Day 15 relative to baseline (*p* = 0.0195). Glycolytic reliance was significantly reduced by Day 15 and remained lower at Day 30 (*p* = 0.0488), indicating decreased dependence on anaerobic or compensatory energy pathways. Baseline respiration and aerobic responsiveness remained stable across timepoints.

Mean Mitochondrial Efficiency increased from 46.9 ± 1.5 to 54.2 ± 4.0 at Day 15 (Cohen's d = 0.76, *p* = 0.021). Glycolytic reliance decreased from 60.2 ± 4.9 to 44.4 ± 3.0 at Day 15 (*d* = −0.70, *p* = 0.047) and remained reduced at Day 30 (43.9 ± 4.4; *d* = −0.78, *p* = 0.047) (Figure 1B). Paired comparisons for all nine bioenergetic parameters at Day 15 and Day 30 are presented in Figures 2B, 3B, respectively.

No increases in oxidative stress markers or mitochondrial network instability were detected, indicating that observed adaptations were not associated with cellular strain or maladaptive stress responses (Figure 4B).

In the athletic cohort, directional changes across mitochondrial efficiency and glycolytic reliance were associated with moderate-to-large within-group effect sizes (Cohen's *d* ≈ 0.70–0.78 across responsive parameters). These adaptations are consistent with optimization of metabolic efficiency and reduced reliance on compensatory glycolytic pathways rather than increased total mitochondrial energy output.

Percentage ranges are reported as approximate relative changes derived from normalized bioenergetic scores across multiple domains and are intended to describe the magnitude of observed trends rather than formal effect sizes.

### 3.2 Subjective fatigue outcome

#### 3.2.1 Chronic fatigue cohort

**Figure 6:**
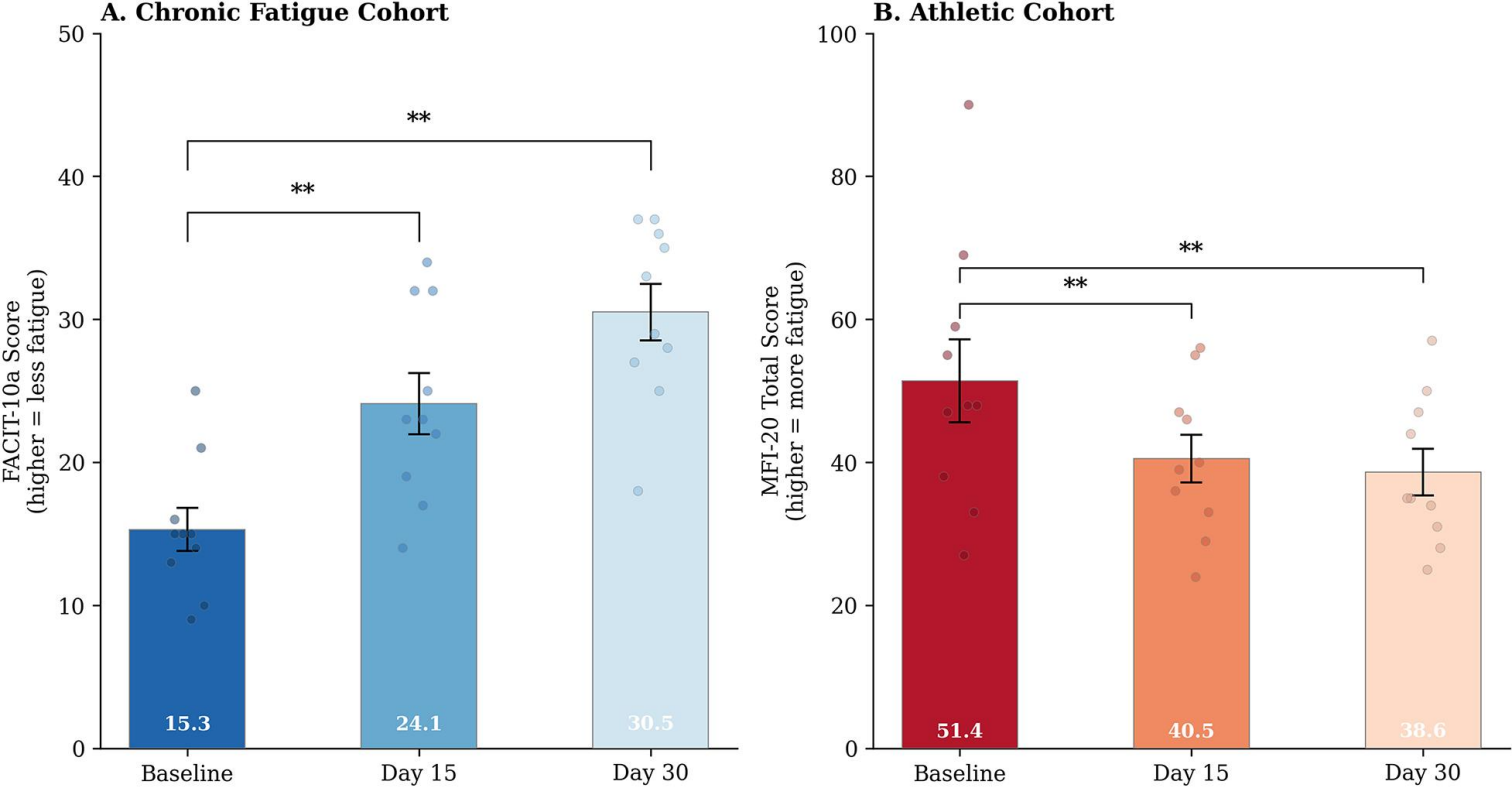
Subjective fatigue scores at Baseline, Day 15, and Day 30. (**A**) FACIT-10a scores in the Chronic Fatigue cohort (higher scores indicate less fatigue); (**B**) MFI-20 total scores in the Athletic cohort (higher scores indicate greater fatigue). Bars represent group means ± SEM. Individual participant scores are shown as circles. Significance brackets indicate Wilcoxon signed-rank test: *p < 0.05, **p < 0.01.

Subjective fatigue assessed using the Functional Assessment of Chronic Illness Therapy–Fatigue short form (FACIT-10a) questionnaire, demonstrated a marked increase in scores over the study period, representing a decrease in overall fatigue. Mean fatigue scores decreased by 15.2 points (−61.5%) from baseline to Day 30. All ten participants showed improvement, with all exceeding the established minimal clinically important difference for the FACIT-10a instrument, indicating changes considered meaningful within the context of the scale. Mean FACIT-10a scores improved from 15.3 ± 1.5 at baseline to 24.1 ± 2.1 at Day 15 and 30.5 ± 2.0 at Day 30 (Figure 6A). Changes from baseline were statistically significant at both Day 15 (*p* = 0.005, *d* = 1.95) and Day 30 (*p* = 0.005, *d* = 2.46).

#### 3.2.2 Athletic cohort

In the athletic cohort, mean total fatigue scores assessed using the MFI-20 decreased by 12.8 points (approximately −24.9%) over the 30-day period. Eight out of ten participants demonstrated improvement. Variability in individual responses appeared consistent with differences in training load and competition schedules during the study period. Mean MFI-20 scores decreased from 51.4 ± 5.8 at baseline to 40.5 ± 3.3 at Day 15 and 38.6 ± 3.3 at Day 30 (Figure 6B). Decreases from baseline were statistically significant at Day 15 (*p* = 0.005, *d* = −1.17) and Day 30 (*p* = 0.005, *d* = −1.33).

#### 3.2.3 Temporal dynamics of change

Across both cohorts, the majority of observed changes occurred early during the study period. Approximately 60%–65% of total observed improvement in both mitochondrial bioenergetic parameters and subjective fatigue scores was evident by Day 15, with continued but more gradual changes through Day 30 (Figure 4).

## 4 Discussion

This exploratory pilot study indicates that daily use of a wearable photobiomodulation device was associated with biologically coherent mitochondrial adaptations, accompanied by parallel changes in perceived fatigue across two physiologically distinct populations. Importantly, observed changes in objective cellular bioenergetics aligned temporally and directionally with validated subjective fatigue measures. This convergence supports the interpretation that the observed patterns reflect physiologically meaningful adaptation rather than isolated reporting effects, while acknowledging the inherent limitations of an uncontrolled exploratory design.

Causal attribution cannot be established because of the single-arm observational study design. The observed changes may also reflect regression to the mean, placebo responses, or variations in training loads; therefore, findings should be interpreted as hypothesis-generating, warranting future randomized controlled study designs. Given the exploratory design and multiple comparisons, the risk of Type 1 error is increased; therefore, findings should be considered hypothesis-generating.

### 4.1 Integration of mitochondrial and subjective outcomes

A key strength of the present study is the convergence of objective mitochondrial bioenergetic measures with subjective fatigue outcomes. Fatigue research has traditionally relied heavily on self-reported instruments which, while clinically informative, provide limited insight into underlying biological processes. In this study, reductions in fatigue scores occurred alongside coordinated directional changes in mitochondrial efficiency, reserve capacity, and metabolic pathway utilization.

Rather than reflecting indiscriminate increases in energy production, the observed bioenergetic adaptations were characterized by improved efficiency, restoration of reserve capacity, and reduced reliance on compensatory glycolytic pathways. This pattern is consistent with optimization of cellular energy handling rather than metabolic overstimulation and supports a mechanistic link between perceived fatigue and mitochondrial energy regulation.

While causality cannot be established, the parallel behavior of biological and subjective measures across timepoints strengthens the biological plausibility of the observed associations and supports further investigation into controlled studies.

### 4.2 Baseline physiology as a determinant of response

Distinct response patterns between cohorts underscore the importance of baseline physiological state in shaping bioenergetic adaptation. Participants with chronic fatigue exhibited recovery-oriented changes, including early improvements in mitochondrial reserve capacity and directional normalization of baseline energy handling. Such responses are consistent with systems operating near energetic constraint at baseline, where restoration of reserve capacity may confer disproportionate functional impact.

In contrast, athletes demonstrated efficiency-driven adaptations without increases in baseline respiration or total energy output. Reductions in glycolytic reliance and improvements in mitochondrial efficiency suggest a shift toward more economical energy utilization rather than increased metabolic throughput. This pattern aligns with expectations in individuals with relatively high baseline mitochondrial function, where optimization rather than recovery is the dominant adaptive pathway.

Collectively, these divergent response trajectories highlight the limitations of uniform interpretations of fatigue and recovery interventions and emphasize the importance of context-specific physiological evaluation when assessing bioenergetic modulation strategies.

### 4.3 Safety, sustainability, and absence of maladaptive stress

A particularly important observation in this study was the absence of increased oxidative stress or mitochondrial network instability across both cohorts. Mitochondrial adaptations that enhance function at the expense of elevated reactive oxygen species production or network fragmentation may yield short-term benefits while potentially increasing longer-term physiological risk (22, 23). In contrast, the observed bioenergetic changes in this study occurred without evidence of increased cellular stress, oxidative burden, or structural instability.

Reactive oxygen generation and mitochondrial network stability data are presented in Figure 2.

Stable mitochondrial network dynamics alongside functional improvements suggest that observed adaptations were supported by maintained organizational integrity. This pattern is consistent with physiologically appropriate bioenergetic modulation rather than stress-induced compensation. Such findings are especially relevant in athletic populations, where excessive suppression of stress signaling or forced upregulation of energy output could negatively affect training adaptation or resilience.

### 4.4 Temporal dynamics of adaptation

The majority of observed changes occurred within the first 15 days of device use, with continued but attenuated changes through Day 30. This temporal pattern suggests an early phase of bioenergetic responsiveness, particularly in systems with lower baseline energetic capacity, followed by stabilization or fine-tuning over time. These dynamics are consistent with models of mitochondrial adaptation in which early improvements reflect relief of energetic bottlenecks, followed by slower regulatory or structural adjustment.

The temporal alignment between changes in mitochondrial bioenergetic parameters and improvements in subjective fatigue further supports a biologically plausible relationship between cellular energy handling and fatigue perception, while not establishing causality.

### 4.5 Implications for photobiomodulation research

Preclinical studies have demonstrated that photobiomodulation can influence mitochondrial respiration, membrane potential, and redox signaling (15); however, human studies directly integrating these mechanisms with functional fatigue outcomes remain limited. The present study contributes to this emerging literature by providing *in vivo* human data that combine mitochondrial bioenergetic assessment with validated subjective outcomes across distinct physiological states.

Importantly, the observed patterns are consistent with photobiomodulation acting as a modulator of energy efficiency and bioenergetic resilience rather than a direct stimulant of energy production. This distinction is relevant for both fatigue-related applications and performance contexts, where sustainability, adaptability, and avoidance of maladaptive stress are critical considerations.

## 5 Limitations and future directions

This study provides initial evidence of an association between photobiomodulation exposure, mitochondrial bioenergetics, and fatigue-related outcomes; however, several limitations warrant consideration.

This study employed a single-arm, exploratory design with a modest sample size, limiting causal inference and generalizability. The absence of a sham-controlled condition precludes definitive attribution of observed changes solely to device exposure.

Additionally, while the mitochondrial assay provides functionally informative bioenergetic data, it reflects peripheral metabolism measured by an adaptive transfer assay and may not fully capture tissue-specific adaptations. While the meScreen™ assay enables minimally invasive, repeatable assessment, it may not fully reflect tissue-specific adaptations.

Given the number of mitochondrial and questionnaire-derived outcome variables analyzed, the statistical power to detect small effects is limited, and effect size estimates should be interpreted with caution. The present findings should therefore be considered exploratory and hypothesis-generating rather than confirmatory. A standardized PBMT protocol was used without evaluation of individualized dose–response relationships. This was intentional, as the study was designed as an observational pilot to establish associative signals. Consequently, factors such as adherence, baseline fatigue, and training load were not systematically analyzed. Future studies incorporating adherence tracking and stratified analyses are needed to define optimal and personalized PBMT dosing strategies.

As this study was designed as an observational pilot to establish associations between PBMT exposure and bioenergetic markers, objective performance testing to complement mitochondrial and subjective fatigue outcomes was not included. While validated fatigue questionnaires provide insight into perceived changes, they do not directly capture functional capacity or performance. Future studies should incorporate standardized physical performance assessments to better determine whether observed mitochondrial adaptations translate into measurable functional improvements.

A lack of post-intervention follow-up limits the assessment of whether observed effects persist after PBMT discontinuation. As an exploratory observational study, the focus was on changes during active use. Future studies should evaluate the durability of mitochondrial and fatigue-related adaptations and define optimal maintenance protocols.

In summary, future studies should incorporate randomized controlled designs, larger cohorts, and extended follow-up periods to assess durability, dose–response relationships, and population-specific effects. Integration with complementary physiological measures, such as autonomic function, performance metrics, or tissue-level assessments, may further clarify systemic implications and underlying mechanisms.

Despite these limitations, the study provides a foundation for further investigation into the role of photobiomodulation in modulating mitochondrial function and fatigue-related outcomes.

## 6 Conclusion

In summary, 30 days of wearable photobiomodulation use was associated with biologically coherent, population-specific mitochondrial adaptations and meaningful reductions in perceived fatigue. Individuals with chronic fatigue exhibited recovery-type bioenergetic improvements, while athletes demonstrated efficiency-oriented optimization without evidence of overstimulation or cellular stress. Although exploratory, the convergence of objective mitochondrial biomarkers and subjective outcomes supports further controlled investigation into photobiomodulation as a candidate modality associated with measurable shifts in cellular bioenergetics in recovery and performance contexts.

## Data Availability

The original contributions presented in the study are included in the article/Supplementary Material, further inquiries can be directed to the corresponding author.

## Supplementary material

Supplementary Table 1 A summary of bioenergetic statistics for both cohorts. Values in red represent statically significant *p*-values.
